# The FGF–FGFR axis as an immune–metabolic rheostat in gastrointestinal inflammation and cancer

**DOI:** 10.3389/fimmu.2026.1805290

**Published:** 2026-03-26

**Authors:** Khamis Salem Saeed Saad, Chunfang Zhou, Guangxin Lu, Gang Wei, Huolong Cha, Ezzaddin Alwabri, Zhifang Zhang, Zequn Sun, Yingying Liao

**Affiliations:** 1 Institute of Deigestive Diseases, Renmin Hospital, Hubei University of Medicine, Shiyan, Hubei, China; 2 Department of Gastroenterology, Renmin Hospital, Hubei University of Medicine, Shiyan, Hubei, China; 3 General Surgery Department, College of Medicine, Ibb University, Ibb, Yemen

**Keywords:** bile acid metabolism, FGFR signaling, fibroblast growth factor, gastrointestinal cancers, inflammatory bowel disease, precision oncology

## Abstract

Fibroblast growth factor (FGF)–fibroblast growth factor receptor (FGFR) signaling constitutes a fundamental regulatory network governing epithelial turnover, metabolic homeostasis, and immune modulation across the gastrointestinal tract. Although discrete FGF pathways have been intensively investigated in inflammatory bowel disease, hepatobiliary disorders, and gastrointestinal malignancies, how these signaling programs are coordinated across pathological contexts remains insufficiently resolved. In this review, we integrate evidence from human cohorts, experimental systems, and clinical studies to conceptualize the FGF–FGFR axis as a context-dependent metabolic-barrier-immune rheostat. Paracrine activation of epithelial FGFR2b supports mucosal restitution and barrier re-establishment following injury, whereas endocrine FGFs—including FGF19, FGF21, and FGF23—couple bile acid signaling, systemic metabolic stress, and mineral balance to intestinal and hepatic inflammatory responses. Perturbation of these adaptive signaling circuits contributes to persistent inflammation and is frequently co-opted by oncogenic events, such as FGFR2b amplification, FGFR2 gene fusions, and aberrant FGF19–FGFR4 activation, during gastrointestinal tumorigenesis. Framing the FGF–FGFR network as an integrated rheostat offers a unifying mechanistic paradigm that links epithelial damage, metabolic dysregulation, and cancer development. It underscores the need for context-selective therapeutic interventions that reconcile tissue repair with long-term oncogenic risk.

## Introduction

The gastrointestinal tract is a unique and highly complex organ system: it absorbs nutrients, excludes pathogens, tolerates a commensal microbiota, and continuously regenerates a single-cell-thick epithelial barrier to maintain tissue homeostasis (1). It achieves this through close integration of epithelial stem cells, mesenchymal niche cells, immune populations, and distant organs such as the liver and kidney, which orchestrate systemic metabolism (2, 3). Because of these compound functions, the FGF family of 22 ligands and their four receptors (FGFR1–4) are prime candidates to coordinate such roles (4, 5). Endocrine ligands require Klotho co-receptors, whereas paracrine ligands often depend on heparan sulfate proteoglycans; Intracrine FGFs also operate in the GI tract (4, 6). FGF7, FGF10, and FGF20 activate epithelial FGFR2b to drive enterocyte proliferation and repair; FGF19, FGF21, and FGF23 are endocrine ligands that sense bile acids, nutrient stress, and phosphate balance; and several other FGFs modulate stromal and immune compartments (6–15).

**Figure 1:**
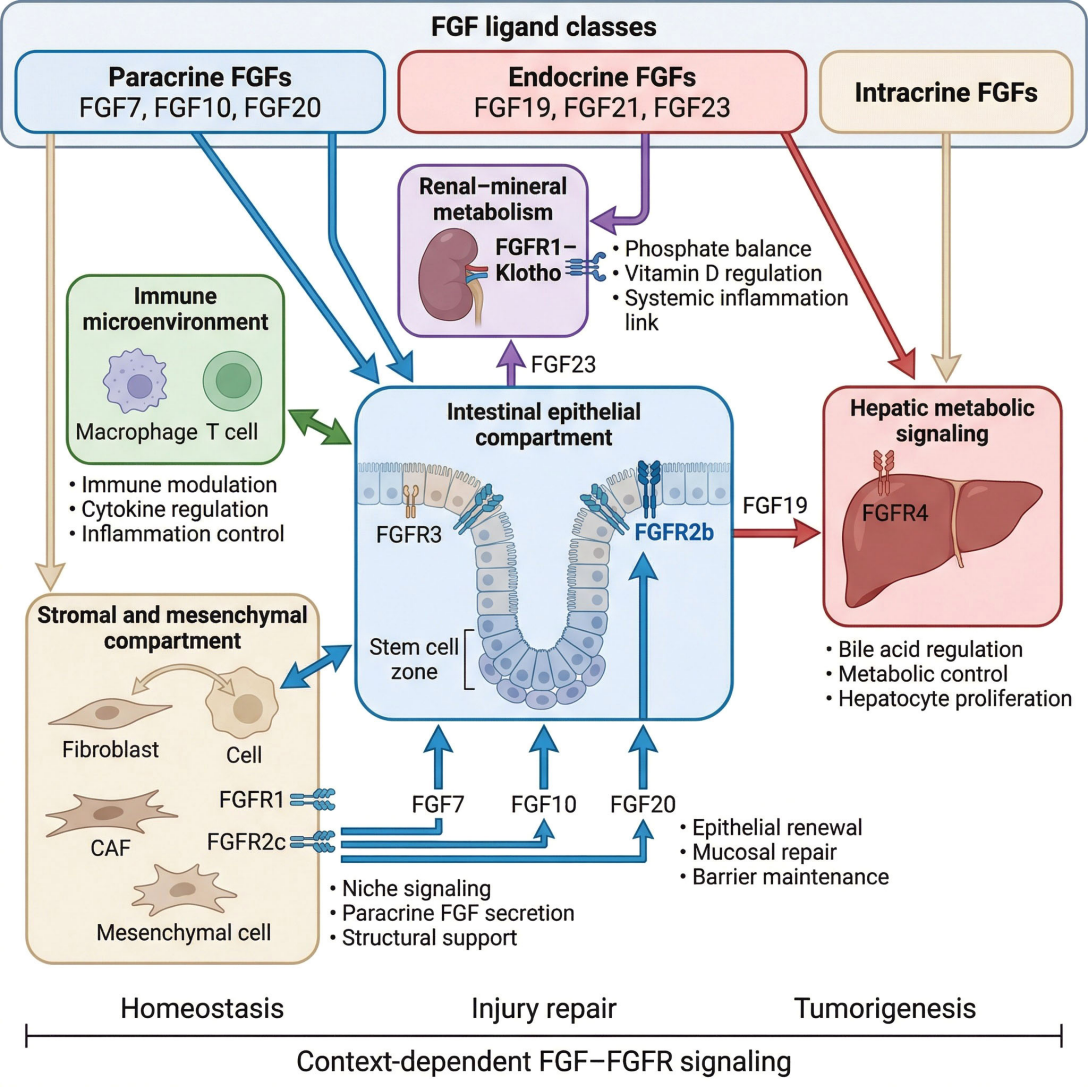
Architectural organization of FGF (fibroblast growth factor)–FGFR (fibroblast growth factor receptor) signaling across the gastrointestinal system. Schematic representation of compartmentalized FGF–FGFR signaling across epithelial, stromal, immune, hepatic, and renal systems. Paracrine Signaling and Niche Support: Paracrine ligands (FGF7, FGF10, FGF20) are secreted by mesenchymal cells and cancer-associated fibroblasts (CAFs) within the stromal compartment. These ligands primarily activate epithelial FGFR2b to drive homeostatic renewal, mucosal repair, and barrier maintenance. Endocrine and Metabolic Integration: The intestinal epithelial compartment functions as a central hub, secreting FGF19 (acting on hepatic FGFR4) to regulate bile acid and metabolic control, and FGF23 (acting on renal FGFR1–Klotho complexes) to maintain phosphate balance and Vitamin D regulation. Immune Microenvironment: The system maintains bidirectional crosstalk with macrophages and T cells to regulate cytokine signaling and systemic inflammation. The Rheostat Gradient: The bottom axis illustrates how these integrated pathways maintain homeostasis during normal physiology but shift toward tumorigenic signaling under chronic dysregulation.

Existing reviews have generally focused on individual aspects, for instance, Inflammatory bowel disease (IBD), liver disease, or FGFR-targeted therapies in cancers (6, 7, 16–23). However, clinical observations suggest that these programs are tightly interconnected. Chronic kidney disease (CKD) is associated with elevated FGF23 and increased GI inflammation; IBD flares alter bile acid–FGF19 signaling; and oncogenic FGFR2 and FGF19 lesions in GI tumors often arise in chronically inflamed tissues (8, 13, 21, 24–29). In this review, we integrate these strands into a single framework: the FGF–FGFR axis as a metabolic–barrier–immune rheostat. We first describe the architectural organization of FGF–FGFR signaling across epithelial, stromal, hepatic, and renal compartments. We then address its roles in inflammatory bowel disease and gastrointestinal cancers in a context-dependent manner. Finally, we outline a therapeutic roadmap and translational priorities to manipulate this axis safely (4–7, 12, 16, 21–23). **Figure 1** illustrates the compartmentalized organization of FGF–FGFR signaling across epithelial, stromal, immune, hepatic, and renal systems.

This framework differs from the traditional view of the FGF–FGFR axis as a linear signaling pathway. Rather than functioning as a static ligand–receptor cascade, the FGF–FGFR network operates as a context-dependent regulatory switch whose biological output depends on temporal duration, ligand composition, receptor isoform distribution, and microenvironmental cues (6–8, 18, 23, 27). The “switching” behavior is determined by coordinated changes in ligand availability, alternative splicing (e.g., FGFR2b versus FGFR2c), co-receptor modulation, and downstream signal biasing between transient ERK-mediated repair and sustained PI3K–AKT, STAT3, or NF-κB activation (18, 23, 34, 42–45). In this model, physiological repair and pathological persistence represent regulated states along a continuum rather than binary on/off conditions.

## Architectural and mechanistic organization of FGF–FGFR signaling in the GI tract

At the level of tissue architecture, the FGF–FGFR system is compartmentalized across diverse cell types. FGFR2b is predominant in epithelial cells of the stomach and intestine, where it responds to subepithelial fibroblast- and niche cell-derived ligands, particularly FGF7, FGF10, and FGF20 (8–10, 30–32). In contrast, the mesenchymal and stromal compartments predominantly express FGFR2c and FGFR1, whereas FGFR3 and FGFR4 are found in selected epithelial, hepatic, and immune subsets (4–8, 30, 31). Developmental studies suggest a handover from FGF10-driven organogenesis to FGF7- and FGF20-mediated adult repair. Fgf10 knockout mice die at birth owing to gastric and intestinal hypoplasia, reflecting an early role in epithelial expansion and patterning. In contrast, FGF7 and FGF20 act as injury-inducible mitogens that support crypt survival and the repopulation of chemically or radiation-damaged gut epithelium through epithelial migration and re-epithelialization (10, 12, 30, 32–35).

Endocrine FGFs extend this framework in several essential ways. FGF19 is induced by bile acid-activated FXR in ileal enterocytes and signals through hepatic FGFR4 to inhibit bile acid synthesis and modulate hepatobiliary metabolism (7, 18, 20, 24, 25, 27–29). FGF21 is secreted in response to metabolic stress and signals via FGFR1–Klotho complexes in adipose tissue and other organs, whereas phosphate and vitamin D homeostasis are regulated by FGF23, produced primarily by osteocytes and signaling through FGFR1–Klotho in the kidney (6, 8, 13–16, 21, 26). Collectively, these ligands connect intestinal activity with systemic metabolism and mineral homeostasis (6–8, 30). This illustrates how FGF10–FGFR2b signaling governs embryonic GI organogenesis, whereas FGF7 and FGF20 assume dominant roles in adult mucosal repair following injury. **Table 1** summarizes the key ligands, receptors, genotype–phenotype relationships in gene manipulation models, and associated human diseases.

**Table 1:** Key FGF ligands relevant to GI biology.

Ligand	Receptor(s)	Gene manipulation phenotype	Human disease association
FGF7	FGFR2b	Knockout → impaired epithelial repair after injury	Reduced expression in subsets of active IBD
FGF10	FGFR2b	Knockout → gastric and intestinal hypoplasia	Congenital GI malformations
FGF20	FGFR2b	Overexpression → enhanced mucosal repair	Upregulated in reparative mucosal states
FGF19	FGFR4	Transgenic overexpression → hepatocarcinogenesis	↓ in IBD; amplification in HCC
FGF21	FGFR1–Klotho	Stress-induced elevation in metabolic organs	↑ in active IBD and metabolic syndrome
FGF23	FGFR1–Klotho	Knockout → hyperphosphataemia, ectopic calcification	↑ in CKD; implicated in systemic inflammation

↑ indicates increased expression and ↓ indicates decreased expression.

Recent phase III clinical trial data further validate FGFR inhibition as a clinically actionable strategy in molecularly defined cancers. In particular, the THOR phase III trial evaluating the selective FGFR inhibitor erdafitinib demonstrated improved overall survival in patients with FGFR3-altered urothelial carcinoma compared with chemotherapy. Although FGFR3 alterations are relatively uncommon in most gastrointestinal malignancies, these findings underscore the principle that effective FGFR-targeted therapy requires precise genomic stratification and context-dependent molecular addiction. The success of isoform-selective FGFR inhibition in non-gastrointestinal cancers strengthens the translational rationale for biomarker-guided FGFR targeting in gastrointestinal tumors (36, 37).

While paracrine and endocrine FGFs act through cell-surface FGFRs, the intracellular FGF subfamily (FGF11–14), also termed iFGFs, operates via receptor-independent mechanisms (4, 6). In the gastrointestinal tract, these intracellular ligands primarily regulate the excitability and trafficking of voltage-gated sodium channels and may influence enteric nervous system signaling and smooth muscle contractility (4, 6). Although their direct involvement in gastrointestinal inflammation and tumorigenesis remains incompletely defined, emerging evidence suggests that iFGFs participate in cellular stress responses and may contribute to the basal homeostatic set point upon which canonical FGF–FGFR signaling exerts its context-dependent effects (6, 7).

## FGF signaling in inflammatory bowel disease

**Figure 2:**
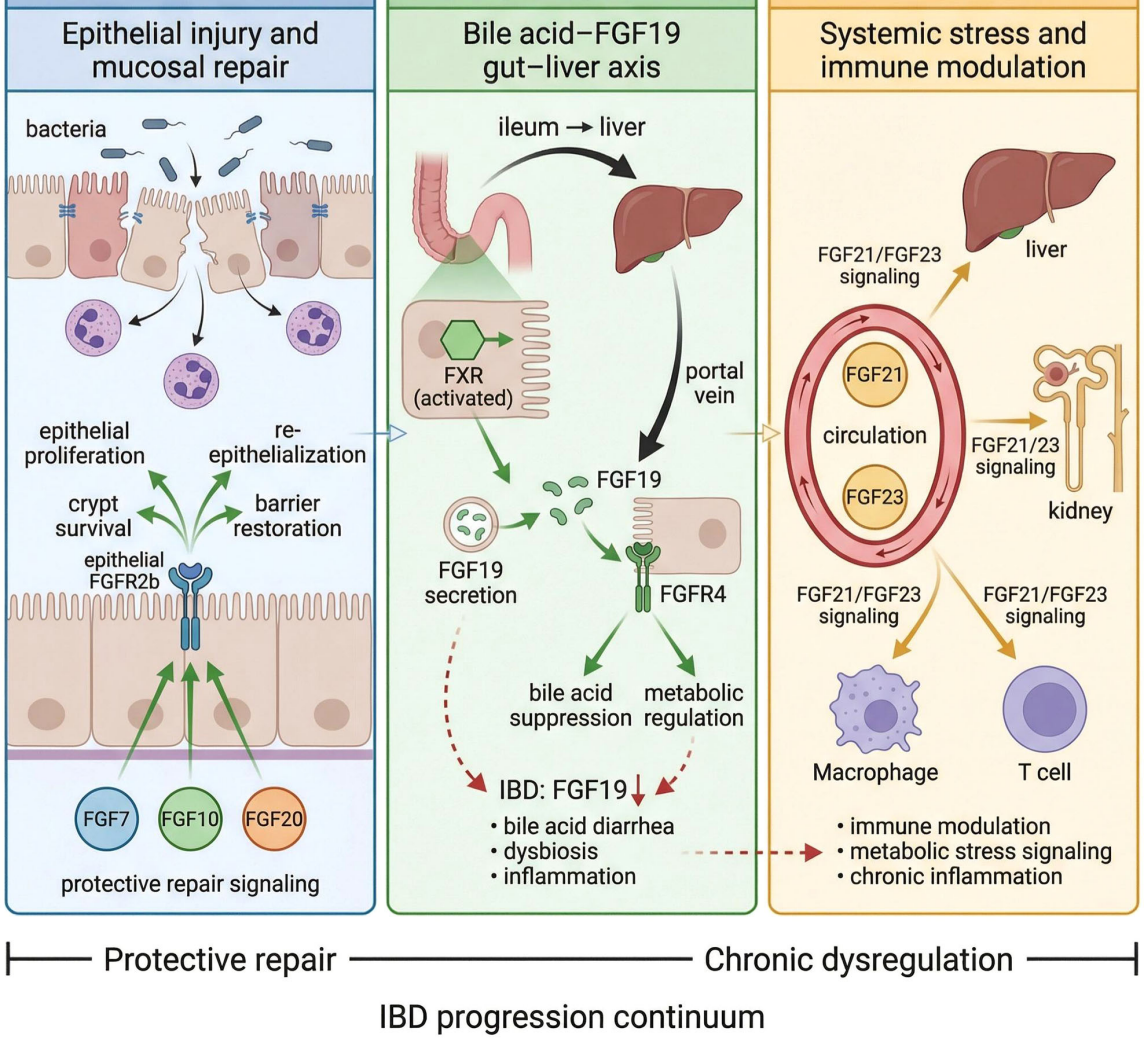
Progression continuum of FGF–FGFR signaling in IBD. Schematic overview of the FGF–FGFR-mediated regulatory networks and their failure during IBD progression. Module 1: Acute Repair (Protective): Following bacterial-induced epithelial injury, paracrine FGF7, FGF10, and FGF20 activate epithelial FGFR2b, triggering ERK-dependent crypt survival, epithelial proliferation, and re-epithelialization. Module 2: Gut–Liver Metabolic Feedback: Activation of FXR in the ileum induces FGF19 secretion, which travels via the portal vein to activate hepatic FGFR4, suppressing bile acid synthesis. In IBD, a reduction in FGF19 (red dashed arrow) leads to bile acid diarrhea, dysbiosis, and amplified inflammation. Module 3: Systemic Stress and Immune Modulation: Systemic endocrine FGF21 and FGF23 levels rise in response to chronic inflammation, modulating macrophage and T-cell activity within the systemic circulation to manage metabolic and inflammatory stress.

Inflammatory bowel disease (IBD) represents a stringent stress test for the FGF–FGFR rheostat in **Figure 2**. Acute flares involve epithelial denudation, barrier breach, dysbiosis, exuberant immune activation, and a high demand for mucosal healing and the re-establishment of barrier integrity to maintain remission (27, 32, 34, 38). While these ligands are protective in acute injury models, sustained or dysregulated activation of epithelial FGFR2b signaling under chronic inflammatory conditions may, in theory, promote excessive proliferation and lower the threshold for dysplastic transformation (18, 19, 23, 42). Thus, the reparative benefits of FGF7/10 must be interpreted within the temporal and inflammatory context in which signaling occurs. In experimental colitis models, exogenous FGF7 or FGF10 improves histological damage scores, crypt survival, and re-epithelialization (10, 12, 30, 32–35). Key FGF–FGFR pathways involved in epithelial repair, gut–liver signaling, and systemic immune–metabolic regulation in IBD are summarized in **Figure 2**.

Genetic disruption of this axis impairs repair and predisposes animals to chronic inflammation (9, 27, 31, 34, 39). Furthermore, FGF genes expected to promote repair are significantly reduced in selected patient subsets with refractory IBD, although the human data remain heterogeneous (10, 27, 30, 32, 34, 38, 40). IBD also couples local mucosal repair to bile acid and FGF19 physiology. Active ileal Crohn’s disease is associated with reduced FGF19 production, resulting in bile acid malabsorption and secretory diarrhea (17, 18, 20, 25, 27, 28). Circulating FGF19 levels are low in this setting, and plasma FGF19 is considered a potential marker of bile acid diarrhea and dysfunction of the FXR–FGF19 axis (17, 20, 25, 28, 29). The gut microbiota represents an additional regulatory layer within the FGF–FGFR rheostat. This interaction is fundamentally bidirectional. On one hand, FGF19-mediated regulation of bile acid synthesis shapes the luminal bile acid pool, thereby influencing microbial composition and metabolic activity (17, 20, 27–29, 39). On the other hand, microbial-derived metabolites—including short-chain fatty acids (SCFAs) and secondary bile acids—modulate FXR activation and fine-tune ileal FGF19 production, linking microbial ecology to systemic metabolic signaling (27, 28, 39). Disruption of this bile acid–microbiota–FGF feedback loop contributes to the dysbiosis-associated inflammatory milieu observed in IBD. Loss of protective commensals and altered metabolite profiles may impair FGF-mediated barrier repair and sustain inflammatory activation (27, 32, 34, 36). In this manner, the microbiota functions as a dynamic metabolic sensor that recalibrates the baseline position of the mucosal rheostat during chronic inflammation.

Therapies that alter bile acid pools or FXR activity can indirectly modulate FGF19-mediated gut–liver communication (18, 20, 25, 27–29, 41). Systemic stress in inflammatory bowel disease (IBD) is also reflected in endocrine FGFs. FGF21 is frequently elevated during active disease and correlates with inflammatory and metabolic markers, suggesting a maladaptive or context-dependent stress response (14, 16, 26, 42). FGF23 is elevated in CKD and may contribute to systemic inflammation with intestinal involvement, raising the possibility of a CKD–FGF23–gut axis that aggravates GI disease in predisposed individuals (8, 13, 15, 21, 26, 43). FGF ligands also reprogram mucosal immunity, particularly within the macrophage and T-cell compartments, thereby shaping the inflammatory tone of the GI microenvironment (6, 7, 16, 21, 23, 27, 32, 40).

**Figure 3:**
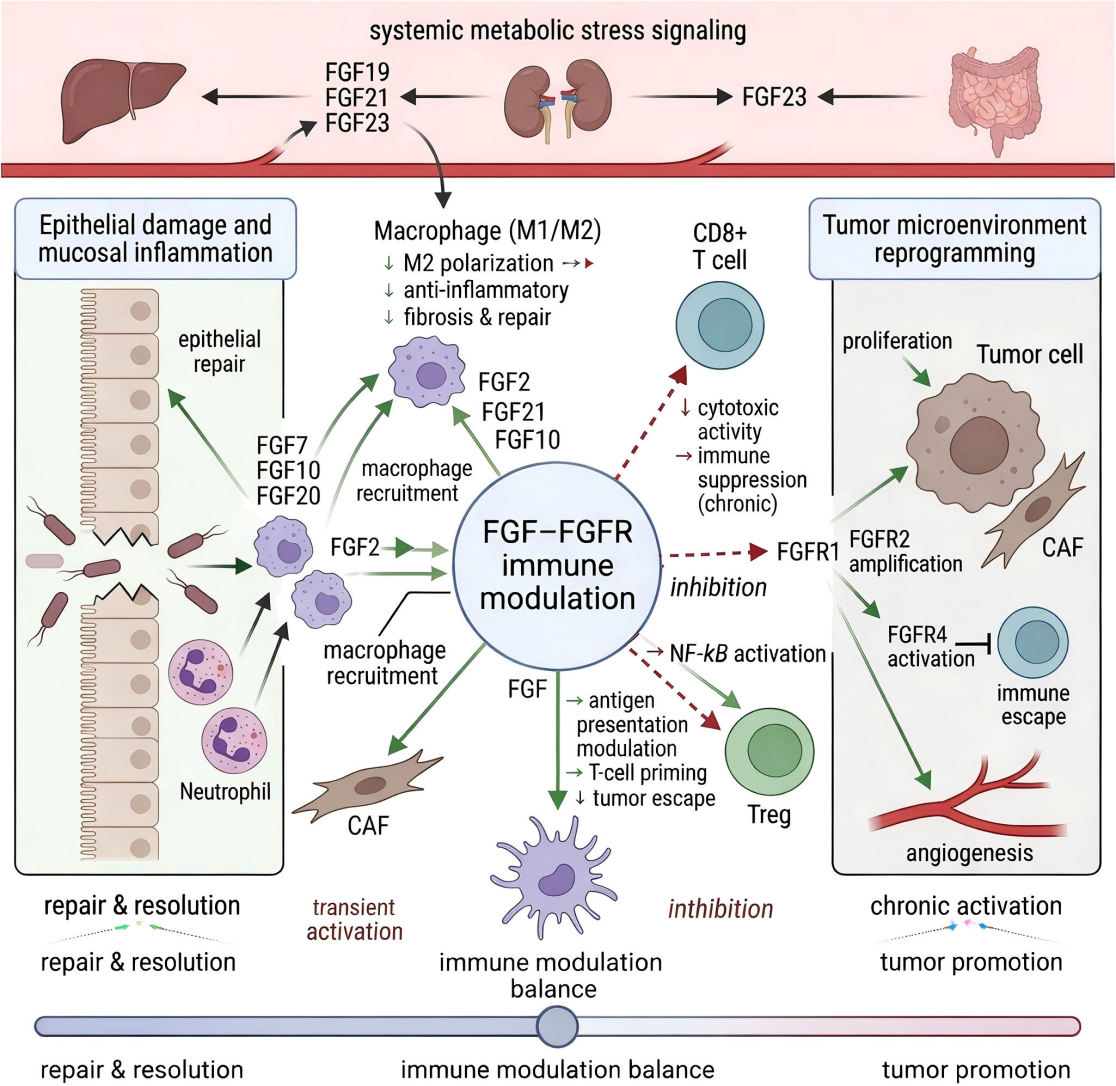
The FGF–FGFR axis as a context-dependent immune–metabolic rheostat in gastrointestinal inflammation and tumorigenesis. Dual-panel representation of time-dependent signaling polarity. Left panel (acute repair phase): Paracrine FGFs activate FGFR2b (green arrows), driving ERK-mediated epithelial restitution and promoting macrophage M2 shift, reducing NF-κB activity, and controlling cytokine production. Endocrine FGFs integrate gut–liver–kidney metabolic signals that fine-tune immune tone. Right panel (chronic activation/tumorigenesis): Sustained FGFR2 amplification or FGF19–FGFR4 activation leads to persistent PI3K–AKT, STAT3, and NF-κB signaling (red arrows), epithelial–mesenchymal transition (EMT), angiogenesis, and immune suppression. Tumor-associated macrophages (TAMs) and regulatory T cells (Tregs) reinforce inhibitory immune circuits (black inhibitory bars). The bottom gradient illustrates rheostat polarization from adaptive repair to oncogenic persistence. Green arrows, activation/pro-reparative signaling Red arrows, sustained oncogenic activation Black inhibitory bars, suppression/feedback inhibition.

These integrated immune–metabolic interactions are summarized in **Figure 3**. FGF2 influences the pattern of antigen-presenting cell and T-cell infiltration; endocrine FGFs such as FGF21 exhibit context-dependent effects ranging from pro-resolution macrophage polarization to suppression of CD8^+^ T-cell effector function (6, 7, 16, 21, 27, 32, 40, 42). These data position FGFs not only as metabolic and epithelial regulators but also as active immunomodulators in the intestinal lamina propria (7, 8, 16, 21, 27, 32, 34, 38, 40). **Table 2** summarizes human and animal evidence spanning epithelial repair, the gut–liver axis, and systemic stress, and classifies each FGF pathway as likely protective, risky, or context-dependent in the IBD setting (10, 14, 17, 21, 25–27, 30, 33, 34, 43). These relationships across three modules: epithelial repair, the gut–liver axis, and systemic stress, involve FGFs. For clinicians, three messages emerge:

**Table 2:** Evidence for FGF pathways in IBD (human and animal studies).

FGF axis	Study type	Main finding	Interpretation	Representative evidence
FGF7/FGF10/FGF20–FGFR2b	Animal model	Exogenous FGF7/FGF10 improves colitis histology and survival	Likely protective (repair programme)	FGF7 or FGF10 rescue in DSS/TNBS colitis models
FGF7–FGFR2b	Human biopsy	Reduced epithelial FGF7 in refractory IBD	Impaired repair capacity	Small cohort biopsy series
FGF19–FXR–FGFR4	Human cohort	Low FGF19 in active ileal CD with bile acid diarrhoea	Risky (bile acid-driven inflammation and diarrhoea)	Bile acid malabsorption and FGF19 studies
FGF21	Human serum	Elevated FGF21 in active IBD correlates with CRP and metabolic markers	Stress response; context-dependent	Serum FGF21 observational studies
FGF23	CKD–IBD link	Elevated FGF23 in CKD is associated with systemic and possibly intestinal inflammation	Context-dependent (CKD–gut axis)	CKD cohorts with inflammatory phenotyping

1. Epithelial repair FGFs are protective in the short term but may entail longer-term oncogenic risk if chronically reactivated.
2. Low FGF19 levels in active IBD are both a biomarker and a potentially modifiable component of bile acid–driven symptoms.
3. High FGF21 and FGF23 levels must be interpreted in the context of systemic metabolic stress and kidney function (2, 6–8, 13–16, 25–27, 43, 44). The progression from chronic inflammatory disorders such as IBD to established gastrointestinal malignancy represents a gradual shift rather than a binary transition (10, 12, 18, 19). FGF pathways that mediate epithelial restitution under inflammatory stress may, when persistently activated or genetically altered, contribute to tumorigenic remodeling of the tissue microenvironment (18, 23, 42–45). We therefore next examine the major actionable FGF–FGFR alterations that define distinct molecular subtypes of gastrointestinal cancers.

## Actionable FGF–FGFR programmers in gastrointestinal cancers

**Figure 4:**
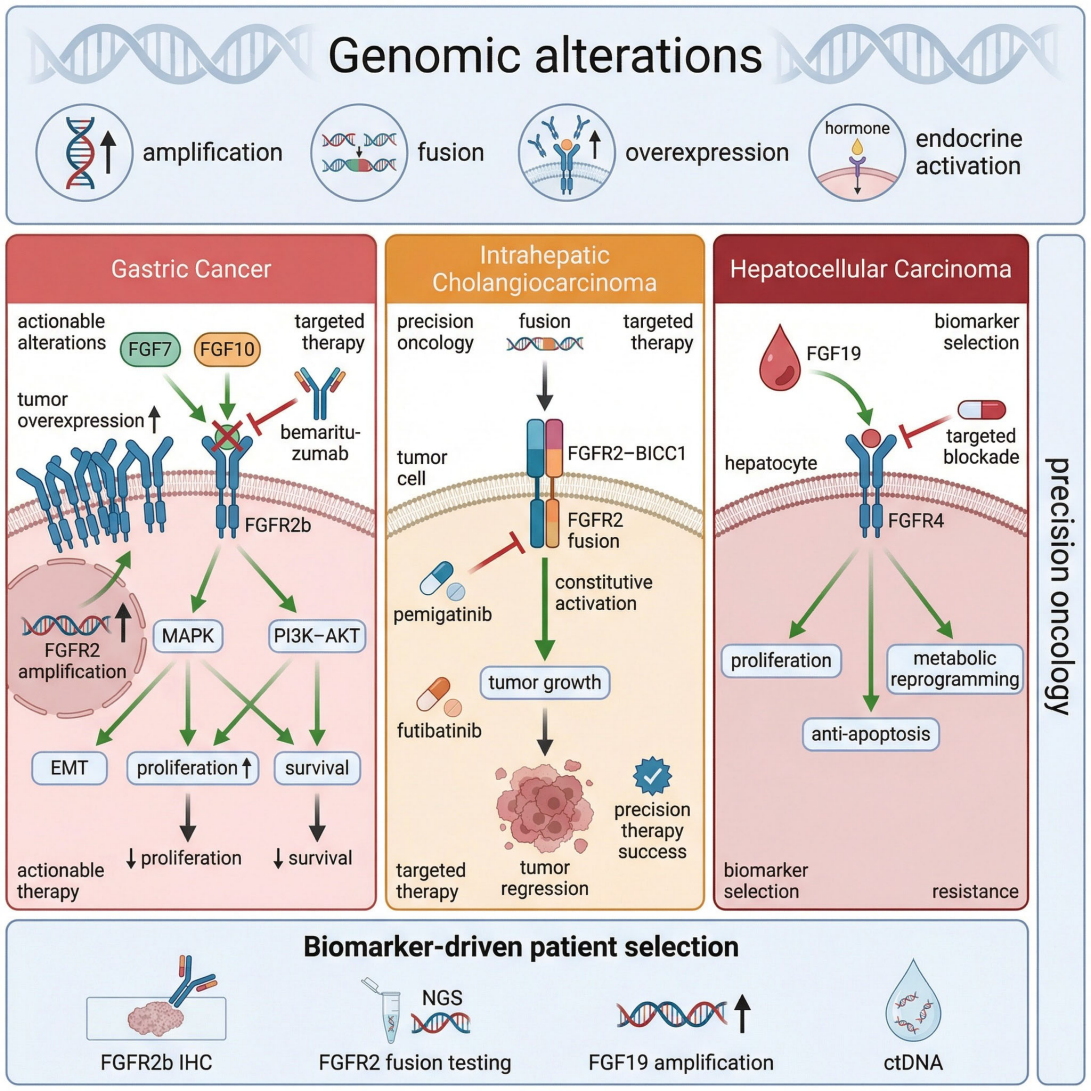
Molecular profiling and precision oncology targets in GI cancers. Mechanistic summary of significant genomic alterations and downstream signaling cascades. Gastric Cancer: FGFR2 amplification drives overexpression and constitutive activation of MAPK and PI3K–AKT pathways, promoting EMT and survival; FGFR2b-specific antibodies inhibit this. Intrahepatic Cholangiocarcinoma: Recurrent FGFR2–BICC1 fusions lead to ligand-independent, constitutive activation and tumor growth, which can be reversed (tumor regression) by selective inhibitors like pemigatinib. Hepatocellular Carcinoma: Endocrine activation of FGFR4 by FGF19 (derived from focal amplification) drives metabolic reprogramming and hepatocyte anti-apoptosis. Biomarker-Driven Selection: Implementation of NGS, IHC, and ctDNA testing is essential for identifying actionable alterations and monitoring therapeutic resistance.

Major FGF–FGFR genomic alterations and corresponding targeted therapeutic strategies across gastrointestinal cancers are summarized in **Figure 4**. Increased bile acid secretion, chronic inflammation, impaired bile acid signaling, and abnormal mucosal repair create a permissive environment for the emergence of tumor-promoting FGF–FGFR signaling axes in the gastrointestinal tract (6, 7, 18, 19, 22–24, 27–29, 32). Three tumor types highlight distinct but interconnected mechanisms: gastric cancer, intrahepatic cholangiocarcinoma, and hepatocellular carcinoma (18, 19, 22–24, 27, 45). In gastric cancer and gastro-esophageal junction adenocarcinoma, FGFR2b gene amplification or overexpression in tumor epithelium drives ligand-dependent oncogenic processes, including epithelial proliferation, survival, and epithelial–mesenchymal transition (EMT) (18, 19, 23, 42, 45–48). Phase II trials of bemarituzumab, a monoclonal antibody targeting FGFR2b, have demonstrated activity against FGFR2b-positive gastric tumors and support FGFR2b as a predictive biomarker (41, 42, 45–47, 49). In intrahepatic cholangiocarcinoma, recurrent FGFR2 fusions generate constitutively active chimeric receptors that are exquisitely sensitive to ATP-competitive FGFR inhibitors (18, 19, 23, 50, 51). Agents such as pemigatinib and futibatinib have shown durable responses in patients with FGFR2 fusion–positive disease, making this one of the most compelling examples of an actionable FGFR alteration in GI oncology (18, 19, 22, 23, 50, 51).

Hepatocellular carcinoma exemplifies an endocrine FGF-driven malignancy. Building upon the gut–liver metabolic circuits described above, focal amplification of FGF19 and consequent overactivation of the FGF19–FGFR4 axis promote hepatocyte hyperproliferation, apoptosis resistance, and metabolic reprogramming (18, 19, 22, 24). In contrast to the transient physiological feedback observed in homeostasis (17, 20), this sustained signaling establishes a mitogenic and survival program that is therapeutically targetable in biomarker-enriched cohorts (22, 24). However, beyond recurrent genomic alterations, emerging evidence implicates FGF–FGFR signaling in the mesenchymal (CMS4) subtype of colorectal cancer, characterized by stromal activation, TGF-β signaling, and poor prognosis (18, 19, 49–51). In this context, paracrine FGF ligands secreted by cancer-associated fibroblasts may enhance tumor cell survival, invasion, and epithelial–mesenchymal transition by activating non-mutational FGFR signaling (49–51). Moreover, stromal–epithelial crosstalk involving FGF signaling has been linked to resistance to anti-EGFR therapies and to immune exclusion within the tumor microenvironment (18, 43, 50). These findings suggest that, in CRC, FGF–FGFR activity may function more as a microenvironmental amplifier rather than a primary oncogenic driver, expanding therapeutic considerations beyond mutation-based stratification. Rare fusions or amplifications may nonetheless be amenable to precision oncology approaches with FGFR inhibitors (18, 19, 22, 23). Emerging germline data further suggest that variability in FGF/FGFR genetics contributes to inter-individual differences in inflammatory and tumor phenotypes (6, 7, 21, 46, 52–54). For example, haplotypes involving the FGFR4 Gly388Arg polymorphism have been associated with colorectal cancer risk and adverse clinicopathological features (21, 46, 52–54). Beyond recurrent GI malignancies, data on FGF/FGFR polymorphisms and GI disease susceptibility are sparse. Still, existing findings support a model in which inherited variation subtly tunes the basal activity of the FGF–FGFR axis (6, 7, 21, 46, 52–54). **Table 3** summarizes major GI tumor types with FGFR alterations, corresponding targeted agents, and the strength of the predictive evidence (17–19, 22–24, 43–47, 50).

**Table 3:** Actionable FGFR alterations in primary GI cancers.

Tumour type	FGFR alteration	Therapeutic agent(s)	Predictive value
Gastric cancer/GEJ	FGFR2b amplification or overexpression	Bemarituzumab; other FGFR2b-directed antibodies	Predictive of benefit in biomarker-selected patients
Intrahepatic cholangiocarcinoma	FGFR2 fusions (e.g. FGFR2–BICC1)	Pemigatinib, futibatinib, infigratinib	Strongly predictive; clear oncogenic driver
Hepatocellular carcinoma	FGF19 amplification; FGF19–FGFR4 activation	Selective FGFR4 inhibitors (e.g., fisogatinib)	Predictive in enriched cohorts; resistance mechanisms emerging
Colorectal cancer	Rare FGFR amplifications/fusions	Exploratory FGFR inhibitors	Unclear; case-by-case precision oncology

## Therapeutic modulation of the FGF–FGFR rheostat: balancing repair and oncogenic risk

**Figure 5:**
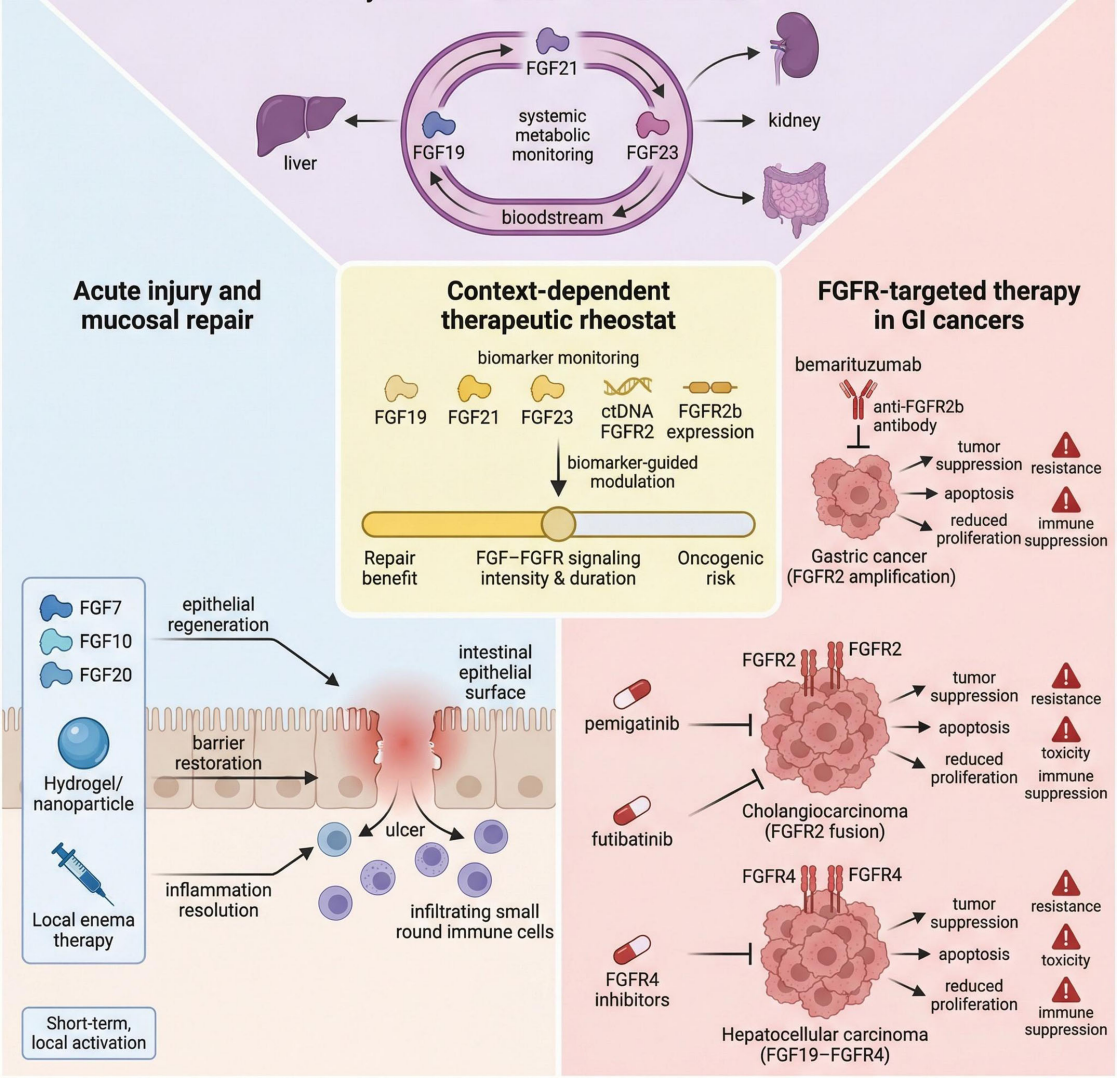
Context-selective therapeutic strategies and the clinical risk–benefit rheostat. This article provides an overview of precision targeting strategies designed to navigate the reparative–oncogenic axis. Inflammatory Setting (Acute/Local): Use of short-term, locally delivered FGF-based therapies (e.g., hydrogels, enemas) to resolve ulcers and restore barrier function without inducing systemic risks. Oncogenic Setting (Targeted Inhibition): Selective blockade of aberrant pathways using antibodies (e.g., bemarituzumab for FGFR2b) or small molecule inhibitors (e.g., pemigatinib, futibatinib) to induce apoptosis and reduce proliferation in gastric, cholangiocarcinoma, and HCC cohorts. Therapeutic Rheostat (Central Node): A biomarker-guided switch (monitoring ctDNA, FGF19/21/23 levels) ensures signaling intensity and duration are optimized to maximize repair benefit while minimizing oncogenic risk and resistance.

The dual nature of FGF–FGFR signaling—its reparative potential in acute injury and its oncogenic potential when aberrantly activated—poses a significant challenge for drug development aimed at modulating this rheostat (7, 8, 10, 16, 18, 19, 21, 22, 24, 27, 30, 34). Context-selective therapeutic strategies targeting the FGF–FGFR axis are summarized in **Figure 5**. Conceptually, therapies focused on epithelial repair occupy one end of the risk–benefit spectrum, whereas anti-tumor strategies based on chronic systemic FGFR inhibition occupy the other (6–8, 16, 18, 19, 21–24, 27). At the “repair” end, short-course, locally delivered FGF agonists are being explored for acute mucosal injuries, such as severe IBD flares or radiation-induced enteritis. Examples include recombinant FGF7, FGF10, or FGF20 delivered via hydrogels, nanoparticles, or enemas to achieve targeted mucosal exposure restricted to damaged areas (10, 12, 27, 30, 32–35, 38, 55). Ideally, these regimens would minimize systemic exposure and avoid chronic stimulation of latent neoplastic clones while promoting epithelial regeneration and barrier restoration (6–8, 10, 16, 27, 30, 32, 34, 35, 38, 55). At the “oncology” end, chronic systemic inhibition of oncogenic FGF–FGFR programs uses selective FGFR1–4 tyrosine kinase inhibitors, anti-FGFR2b antibodies, and FGFR4-selective agents (17–19, 22–24, 27, 42, 45–51). Combination regimens with immune checkpoint blockade or DNA-damaging chemotherapy are being tested to overcome resistance and exploit synthetic lethal interactions, such as impaired homologous recombination in FGFR2-driven tumors (17–19, 22–24, 45, 50, 51). These genetic changes identify molecular subgroups that can be targeted with precision therapies against FGFR. The left section illustrates FGF-mediated repair approaches for inflammatory bowel disease and radiation damage, while the right section details treatment strategies targeting FGFR—both alone and in combination—for gastrointestinal cancers (6–8, 10, 16, 18, 19, 21, 23, 24, 27, 30, 32, 34, 50, 51). A central risk–benefit axis emphasizes that biomarkers of repair benefit and oncogenic risk must be monitored, including circulating FGF19, FGF21, and FGF23; tissue FGFR2b expression; and FGFR2 fusions detected in circulating tumor DNA (ctDNA) (6–8, 13–18, 24–27).

## Future directions and translational priorities

Several priorities must be addressed to safely and effectively harness the FGF–FGFR rheostat in GI disease. First, given the sequence similarity among FGFR1–4, particularly in the ATP-binding pocket, first-generation ATP-competitive inhibitors are expected to be cross-paralog and to incur significant toxicities (6, 7, 17–19, 21, 23, 24, 27, 50). True isoform- or splice-variant selectivity requires exploiting subtle structural differences in non-conserved residues within the hinge or activation loop, as illustrated by paralog-specific covalent FGFR4 inhibitors, or targeting unique extracellular domains, as in FGFR2b-selective antibodies. High-resolution structural studies may also reveal conformational preferences and allosteric sites that can be leveraged for selectivity (6, 7, 18, 19, 22–24, 27, 42, 45–51). On the agonist side, an ideal scenario would activate epithelial FGFR2b-dependent repair programs transiently and locally, without chronically stimulating protumorigenic signaling in dysplastic clones (6–10, 16, 27, 30, 31, 34, 35, 38, 55). On the antagonist side, highly selective FGFR2 kinase inhibitors and FGFR2b-directed monoclonal antibodies exemplify complementary design strategies: small molecules that exploit rare sequence differences to achieve paralog bias, and biologics that recognize isoform-specific extracellular epitopes (17–19, 22–24, 27, 42, 45–48, 50, 51, 56). *De novo*-designed mini-proteins and biparatopic antibodies that engage distinct epitope pairs on FGFR2c or fusion proteins offer a third route to isoform-selective blockade and may be particularly useful against resistance mutations within the kinase domain (17–19, 23, 24, 50, 52). A practical translational agenda will require head-to-head evaluation of these modalities in models spanning the continuum from acute mucosal injury to established tumor (7, 16, 18, 21, 23, 27, 34). Second, composite biomarkers integrating systemic ligands, tissue receptor status, and ctDNA will be needed to guide therapy and detect toxicity early (6, 7, 13–15, 17, 21, 24, 45). For example, serum FGF19 levels combined with bile acid profiles and hepatic imaging may distinguish on-target from off-target effects of FGFR4 inhibitors. In contrast, ctDNA assays for FGFR2 fusions or resistance mutations can refine patient selection and monitor disease evolution in cholangiocarcinoma and gastric cancer (6, 7, 17, 18, 22, 24, 25, 27, 45). Measurements of FGF21 and FGF23, together with renal and metabolic parameters and markers of tissue injury, may help distinguish adaptive stress responses from pathogenic chronic activation in patients with overlapping CKD, IBD, and fatty liver syndromes (6, 8, 11, 13–15, 21, 25, 26, 29, 43). Third, next-generation preclinical models should incorporate all relevant cellular ecosystems, including immune components influenced by FGFs (6–8, 16, 21, 27, 32, 34, 38, 40). This can be achieved by co-culturing organoids derived from human intestinal, gastric, or biliary epithelium—engineered to express patient-specific FGFR alterations—with stromal fibroblasts, endothelial cells, and immune subsets such as macrophages and CD8^+^ T cells under defined bile acid and metabolic conditions. These models will be well-suited to test isoform-selective agents, dissect epithelial-intrinsic versus immune-mediated effects, and quantify how modulation of the FGF–FGFR axis affects barrier restoration, cytokine networks, and tumor–immune crosstalk relative to traditional monocultures (7, 8, 16, 18, 21, 22, 27, 32, 34, 38).

Finally, longitudinal studies across disease states from at-risk populations through early inflammation to established cancer tracking FGF ligands, receptor isoforms, germline polymorphisms, and downstream transcriptional programs will be needed to determine whether FGF modulation is preventive, therapeutic, or harmful (6, 7, 16, 17, 19, 21, 45, 52, 53). Ultimately, *in vivo* studies integrating germline FGF/FGFR genotyping with serial liquid biopsies in IBD and GI cancer cohorts may reveal how inherited variation sets the baseline position of the metabolic–barrier–immune rheostat, and how therapeutic interventions shift patients along this axis over time (16, 19, 27, 32, 46, 51, 53).

## Discussion

Conceptualizing the FGF–FGFR network as a rheostat rather than a discrete signaling pathway provides a systems-level framework for understanding gastrointestinal disease progression (6, 7). Unlike linear pathway or axis models that imply binary activation states, a rheostat emphasizes graded, reversible, and context-dependent modulation of biological outputs. This framing is particularly appropriate for the gastrointestinal tract, where epithelial renewal, immune tolerance, and metabolic sensing must be continuously tuned rather than switched on or off (1, 4). A significant strength of the rheostat model is its capacity to integrate heterogeneous observations across disease states. It explains why epithelial FGFR2b signaling driven by FGF7, FGF10, and FGF20 is protective during acute mucosal injury (10, 12, 33). Yet similar signaling programs—when sustained or recontextualized—are implicated in tumorigenesis (18, 19, 23). The model also incorporates endocrine FGFs: bile acid–FXR–FGF19 signaling, metabolic stress–induced FGF21, and mineral metabolism–linked FGF23, which dynamically shift the baseline set point of epithelial and immune responsiveness (17, 20, 26). However, the rheostat model has defined boundaries. It is not designed to predict individual patient trajectories, nor does it fully capture stochastic oncogenic events, clonal evolution, or intratumoral heterogeneity (22, 23). Moreover, disease phenotypes driven primarily by immune dysregulation or microbiota-independent epithelial injury may fall outside its explanatory scope. Thus, the rheostat should be viewed as a systems integrator rather than a universal causal model. Notably, several high-impact studies published within the past two to three years have further refined this systems-level perspective. Advances in epithelial organoid modeling and macrophage–epithelial co-culture systems have provided mechanistic insights into FGF-mediated barrier repair and immune modulation (32, 34, 38, 55). Concurrently, recent clinical characterization of FGFR2b expression in gastric cancer and updated phase II–III data on FGFR-targeted agents have strengthened the translational relevance of isoform-selective strategies (42, 46–48, 54). These developments underscore that the rheostat model is not merely conceptual but is increasingly supported by contemporary experimental and clinical evidence.

The inflammatory bowel disease–cancer continuum represents a biological gradient rather than a dichotomy. Chronic inflammation, repeated epithelial injury, compensatory hyper-repair, and eventual malignant transformation unfold over years to decades (27, 32). Traditional pathway-centric models struggle to capture this continuity, as they tend to assign signaling networks either protective or pathogenic roles depending on disease category.

The rheostat framework explains how the same FGF–FGFR circuitry mediates divergent outcomes depending on duration, intensity, and tissue context. In early or acute IBD, upregulation of epithelial FGFR2b ligands enhances barrier restoration and limits microbial translocation (10, 30). With chronic inflammation, however, sustained FGF-driven proliferation may lower the threshold for oncogenic transformation, particularly in genetically susceptible epithelial clones (18, 19, 43). Systemic modifiers are integral to this continuum. Reduced FGF19 signaling in active ileal disease alters bile acid composition, reshaping epithelial stress and immune activation (17, 20). Concurrent elevations in FGF21 or FGF23 reflect metabolic or renal stress that further shifts immune tone and epithelial vulnerability (14, 26). A single linear axis cannot adequately represent these multilayered effects.

The rheostat model is particularly effective at explaining paradoxical observations. It clarifies why interventions that enhance mucosal repair may simultaneously increase oncogenic risk, why circulating FGFs function as biomarkers without being direct therapeutic targets, and why FGFR inhibition is effective only in molecularly selected tumors (22, 24, 46). It also explains the limited success of non-selective FGFR blockade in unselected inflammatory or oncologic populations.

However, the model does not define quantitative thresholds separating adaptive from maladaptive signaling, nor does it predict when repair-associated proliferation becomes irreversibly oncogenic. Spatial heterogeneity within tumors, where FGFR signaling may be critical in some niches and irrelevant in others, is also insufficiently captured (23, 47). These limitations highlight the need for experimental systems capable of resolving temporal and spatial signaling dynamics.

A central clinical implication of the FGF–FGFR rheostat is the inherent tension between tissue repair and oncogenesis. Short-term activation of FGF signaling may be clinically critical in severe mucosal injury, radiation enteritis, or fulminant colitis (10, 12, 35). In contrast, prolonged or systemic activation of the same pathways may promote tumor initiation or progression (18, 19, 24). This duality is not unique to FGFs. TGF-β and Wnt signaling exhibit similar context-dependent behavior: both are essential for tissue repair and stem cell maintenance, yet contribute to cancer when deregulated (6, 7). What distinguishes FGFs is their integration of local epithelial signals with systemic metabolic cues, linking oncogenic risk to host metabolic and inflammatory states (21, 26).

Clinically, this argues against chronic, non-selective enhancement of FGF signaling in inflammatory disease and cautions against indiscriminate long-term FGFR inhibition in oncology without molecular stratification.

Biomarker-guided, context-selective modulation, therefore, represents the only viable strategy. The rheostat framework implies that therapeutic decisions should focus not on whether to activate or inhibit FGF (fibroblast growth factor) signaling, but on when, where, and in whom to do so. Biomarker-guided modulation is therefore essential. Composite biomarker panels integrating circulating FGF19, FGF21, and FGF23, epithelial FGFR2b expression, and tumor-specific FGFR alterations provide the necessary contextual resolution (17, 26, 40).

Importantly, these biomarkers must be interpreted collectively rather than individually. FGFR inhibitors show robust efficacy only in tumors with apparent FGFR addiction, whereas FGFR2b-directed antibodies succeed because of isoform and tissue specificity (42, 46). Similarly, future FGF-based repair strategies must be short-course, locally delivered, and tightly monitored to minimize oncogenic risk.

Finally, the rheostat model exposes fundamental limitations in current experimental paradigms. The assumption that a single FGFR inhibitor can address diverse gastrointestinal diseases is biologically untenable. It ignores receptor isoform diversity, tissue compartmentalization, and systemic feedback loops (7, 22).

Progress will require integrated experimental platforms combining epithelial organoids with immune and stromal components under defined metabolic conditions. Such models are essential for capturing how FGF–FGFR signaling simultaneously reshapes barrier function, immune tone, and tumor evolution. Without this level of integration, translational efforts risk repeating past failures driven by oversimplified biology (16, 23).

## Conclusion

The FGF–FGFR axis has emerged as a central hub linking metabolism, barrier integrity, and immune regulation within the GI tract. By integrating developmental biology, mucosal repair, bile acid and mineral metabolism, and oncogenic signaling, this review outlines a unifying framework in which FGF–FGFR programs shape trajectories from homeostasis to IBD and GI cancers. The therapeutic potential of modulating this axis is substantial, but so are the risks. Progress will depend on context-selective agents, robust biomarkers, and preclinical models that faithfully recapitulate the underlying physiology. Together, these efforts should enable translation of FGF–FGFR biology into clinically meaningful benefits for patients with GI diseases.
