# Correction: The FGF–FGFR axis as an immune–metabolic rheostat in gastrointestinal inflammation and cancer

**DOI:** 10.3389/fimmu.2026.1841292

**Published:** 2026-04-16

**Authors:** Khamis Salem Saeed Saad, Chunfang Zhou, Guangxin Lu, Gang Wei, Huolong Cha, Ezzaddin Alwabri, Zhifang Zhang, Zequn Sun, Yingying Liao

**Affiliations:** 1 Institute of Deigestive Diseases, Renmin Hospital, Hubei University of Medicine, Shiyan, Hubei, China; 2 Department of Gastroenterology, Renmin Hospital, Hubei University of Medicine, Shiyan, Hubei, China; 3 General Surgery Department, College of Medicine, Ibb University, Ibb, Yemen

**Keywords:** bile acid metabolism, FGFR signaling, fibroblast growth factor, gastrointestinal cancers, inflammatory bowel disease, precision oncology

Author “Khamis Salem Saeed Saad” was erroneously assigned as corresponding author. The correct corresponding authors are “Zequn Sun and Yingying Liao”.

The original version of this article has been updated.

